# Manual dexterity and plaque accumulation in patients with rheumatoid arthritis

**DOI:** 10.3389/froh.2026.1883158

**Published:** 2026-07-22

**Authors:** Luki Astuti, Endang W. Bachtiar, Rudy Hidayat, Boy Muchlis Bachtiar, Benso Sulijaya, Trijani Suwandi, Chia Wei Cheah, Yuniarti Soeroso, Muhammad Ihsan Rizal

**Affiliations:** 1 Doctoral Program, Faculty of Dentistry, Universitas Indonesia, Jakarta, Indonesia; 2 Department of Periodontology, Faculty of Dentistry, Universitas Trisakti, Jakarta, Indonesia; 3 Department of Oral Biology, Faculty of Dentistry, Universitas Indonesia, Jakarta, Indonesia; 4 Rheumatology Division, Department of Internal Medicine, Faculty of Medicine/Cipto Mangunkusumo Hospital, Universitas Indonesia, Jakarta, Indonesia; 5 Department of Periodontology, Faculty of Dentistry, Universitas Indonesia, Jakarta, Indonesia; 6 Department of Restorative Dentistry, Faculty of Dentistry, Unversity of Malaya, Malaysia; 7 Department of Oral Biology, Faculty of Dentistry, Universitas Trisakti, Jakarta, Indonesia

**Keywords:** motor skills, oral hygiene, periodontal diseases, periodontitis, rheumatoid arthritis

## Abstract

**Introduction:**

Rheumatoid arthritis (RA) reduces manual dexterity, potentially hindering effective oral hygiene and increasing periodontal risk. However, this relationship has not been fully clarified.

**Methods:**

This cross-sectional study included 80 participants (40 with RA and 40 without RA), all diagnosed with gingivitis or periodontitis. The groups were recruited from different settings and were not matched for demographic or systemic characteristics. Periodontal parameters were assessed using full-mouth bleeding score (FMBS), plaque score, probing depth (PD), and clinical attachment loss (CAL). Manual dexterity was evaluated using the Moberg Pick-Up Test (MPUT), comprising four subtests: dominant hand eyes open (DHEO), non-dominant hand eyes open (NdHEO), dominant hand eyes closed (DHEC), and non-dominant hand eyes closed (NdHEC). Associations were analyzed using Spearman's correlation, and multivariable analysis was performed adjusting for age and comorbidities.

**Results:**

Periodontitis was more prevalent in the RA group, whereas gingivitis predominated in the non-RA group (*p* = 0.001). The plaque score was significantly different between groups (*p* = 0.036). All MPUT subtests showed significantly poorer performance in the RA group compared with controls (*p* < 0.05). Within the RA group, NdHEO and NdHEC subtests were associated with plaque scores (*p* = 0.006, *p* = 0.005). In multivariable analysis, performance in NdHEC subtest was significantly associated with plaque scores (*p* = 0.036; OR 0.033, 95% CI 0.001–0.797) after adjustment for age and comorbidities.

**Conclusions:**

Manual dexterity, as assessed by the MPUT, was associated with greater plaque accumulation but not with periodontal severity. While these findings suggest a potential link between functional limitations and oral hygiene in RA patients, a causal relationship cannot be inferred due to the cross-sectional design of this study.

## 1 Introduction

RA is a long-term autoimmune inflammatory disease that predominantly affects the synovial membranes of multiple joints, causing persistent inflammation, hyperplasia, and the gradual deterioration of cartilage and bone. If left untreated, RA can lead to severe joint abnormalities, restricting daily activities and lowering patients' quality of life (1, 2). The global prevalence of RA stands at approximately 0.46% (3), exhibiting a higher prevalence in females with a female-to-male ratio of roughly 3:1 (1). RA is induced by a complex confluence of genetic predispositions and environmental factors, including recent suggestions that the human microbiome may serve as a critical trigger for inflammatory arthritis (4, 5). Periodontitis—a chronic, multifactorial inflammatory disease exacerbated by dysbiotic dental biofilm accumulation—deteriorates the supporting periodontal structures and is widely recognized as a bidirectional risk factor for RA (1, 6, 7). Mechanistically, certain periodontal bacteria, such as *Porphyromonas gingivalis* (*P. gingivalis*) and *Aggregatibacter actinomycetemcomitans* (*A. actinomycetemcomitans*), play a pivotal role in triggering autoantibody responses. Specifically, *P. gingivalis* produces the peptidylarginine deiminase (PPAD) enzyme, which uniquely citrullinates C-terminal arginine residues in host proteins, generating neoantigens that drive anti-citrullinated protein antibody (ACPA) production—a hallmark of RA-associated autoimmunity. Simultaneously, *A. actinomycetemcomitans* promotes hypercitrullination in neutrophils through its leukotoxin A (LtxA), disrupting host citrullinating enzymes and generating a diverse array of citrullinated proteins that closely resembles the profile observed in inflamed RA joints. Together, these pathogens provide a compelling mechanistic link between localized oral infection and systemic autoimmunity (6, 8). These processes converge with RA's immune pathways, in which ACPAs, autoreactive lymphocytes, and pro-inflammatory cytokines such as tumor necrosis factor-*α* (TNF-*α*) and interleukin-6 (IL-6) drive systemic inflammation (9).

Numerous studies indicate that individuals with RA exhibit significantly poorer periodontal health, characterized by deeper periodontal pockets and greater bone loss, compared to healthy controls (10–12). Impaired manual dexterity resulting from joint pain and deformity adversely affects daily oral hygiene practices. Joint pain and deformity impair manual dexterity, limiting patients' ability to perform effective tooth brushing and flossing (13). This mechanical failure in biofilm control fosters plaque accumulation and a stagnant microenvironment that promotes dysbiosis, shifting early colonizers toward a pathogenic anaerobic subgingival biofilm (14). Hashimoto et al. confirmed this link, reporting that RA-related functional impairment significantly increases the risk of periodontal inflammation (15).

Manual dexterity is the ability to do things that need precise and coordinated hand and finger motions and encompasses a synthesis of gross motor skills (GMSs) and fine motor skills (FMSs). FMSs include fine and accurate movements, whereas GMSs require more extensive manual skills (16, 17). The Moberg Pickup Test (MPUT**)** is a timed, standardized evaluation used to quantify fine motor coordination in the upper extremities. Originally developed by Moberg, this assessment focuses on the functional dexterity of the hand. This assessment has proven to be a highly reliable and valid tool for evaluating individuals with various hand-related conditions. Specifically, its effectiveness is well-documented in clinical populations affected by carpal tunnel syndrome, RA, and osteoarthritis (18–20). A prior study indicated that manual dexterity was associated with plaque score, which in turn was linked to gingival bleeding (17, 21, 22). Additionally, evidence suggests that patients with RA may exhibit reduced oral hygiene (23). Although the association between RA and periodontal disease is well established, the role of physical functional limitations—particularly manual dexterity—remains insufficiently explored. Therefore, this study aims to investigate whether objectively measured deficits in manual dexterity, as assessed by the MPUT, are associated with plaque accumulation in an understudied Indonesian population.

## 2 Methods

### 2.1 Participants

This cross-sectional study examined and correlated manual dexterity and plaque accumulations in patients with and without RA across two healthcare centers in Jakarta, Indonesia. Ethical approval was obtained from the Ethics Committee of the Faculty of Medicine, Universitas Indonesia – Cipto Mangunkusumo Hospital (registration number 1206), and all participants provided written informed consent prior to the study.

The sample size was calculated using pocket depth (PD) data from a previous study (24). Based on this, a minimum of 68 participants was required to achieve 80% statistical power at a 5% significance level with an effect size of 0.69, as determined using G*Power 3.1 software (Informer Technologies, Düsseldorf, Germany). PD was selected because it represents a stable cumulative measure of periodontal tissue destruction. To account for potential dropouts, 108 participants were initially recruited. After applying inclusion and exclusion criteria, 80 participants remained in the final analysis. A *post-hoc* power calculation based on this sample size and the observed effect size confirmed that the study achieved a statistical power of 0.86, exceeding the originally planned threshold of 0.80.

**Figure 1:**
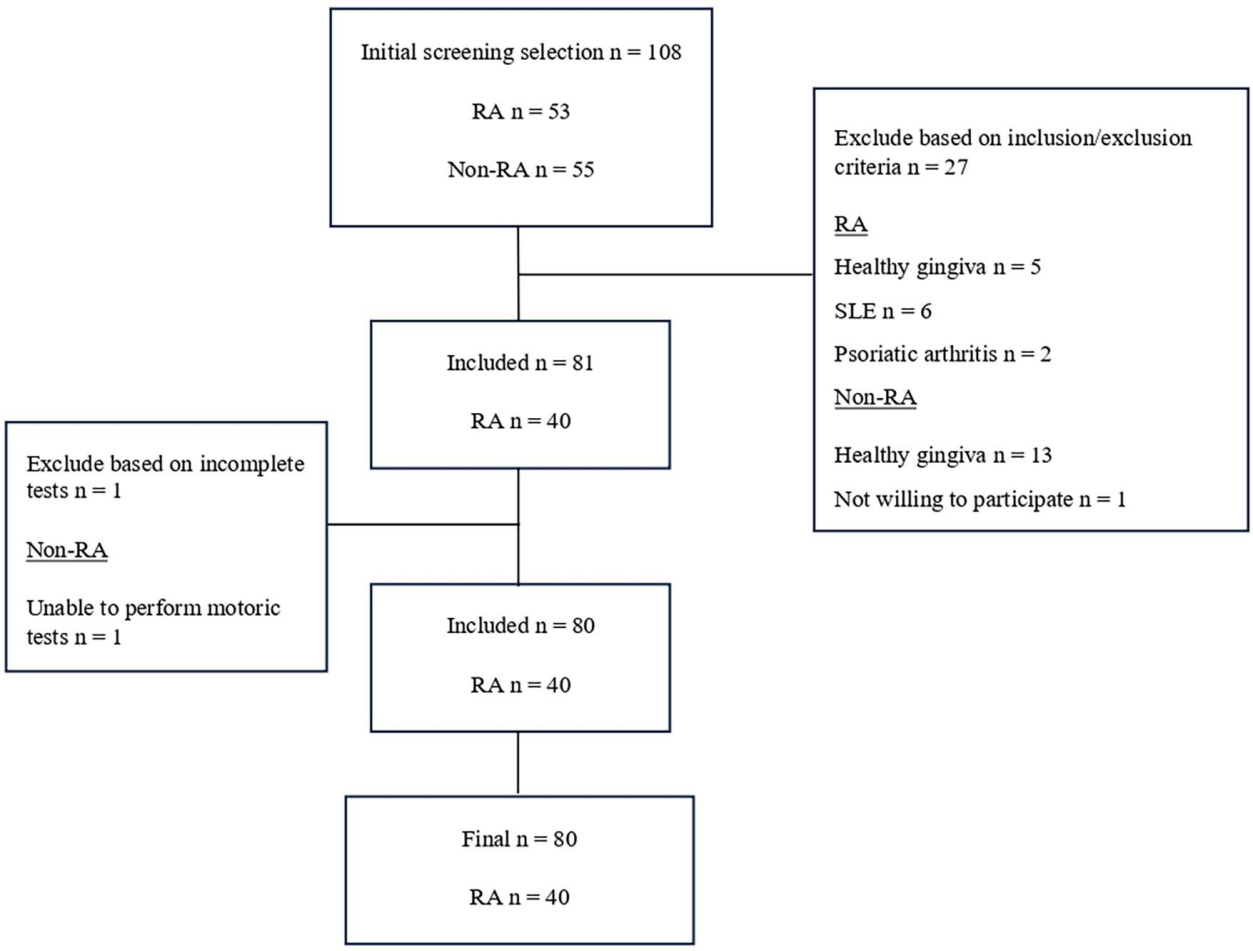
The flowchart of participants inclusion and exclusion criteria*.*

Forty adults with RA were recruited from the National Referral Center, Rumah Sakit Cipto Mangunkusumo (RSCM), Jakarta, Indonesia. RA diagnoses were confirmed by rheumatologists according to the 2010 American College of Rheumatology/European League Against Rheumatism (ACR/EULAR) classification criteria. The comparison group comprised 40 systemically healthy individuals with gingivitis or periodontitis, recruited from the Dental Hospital, Faculty of Dentistry, Universitas Indonesia. RA patients were enrolled from a national referral hospital to ensure standardized diagnostic procedures and assessment of disease activity, whereas the comparison group was drawn from a dental hospital where comprehensive periodontal examinations are routinely performed. Both facilities are located within the same geographic area and serve populations with comparable socioeconomic characteristics and similar access to healthcare. Identical eligibility criteria, periodontal examination protocols, and examiner calibration were applied across both groups to minimize selection and measurement bias. Eligible participants were aged 18–59 years. Exclusion criteria included pregnancy, receipt of periodontal therapy or antibiotics within the previous three months, healthy gingiva, or autoimmune diseases other than RA. In total, 80 participants (40 RA and 40 non-RA) were enrolled between September 2025 and January 2026 (Figure 1).

### 2.2 Periodontal status examination

Prior to the main clinical evaluation, calibration was conducted by a periodontist who assessed the PD of five independent patients across two separate sessions one day apart. Data from these calibration subjects were excluded from the primary study sample. The intra-examiner reliability was confirmed with an Intraclass Correlation Coefficient (ICC) of 0.85. Clinical parameters including PD, recession, CAL, plaque, and FMBS were recorded for both the RA and non-RA groups. PD and gingival recession measurements were obtained at six sites per tooth for all teeth using the UNC-15 periodontal probe. Plaque accumulation was assessed using a full-mouth recording. The presence of plaque was evaluated at six sites per tooth for all erupted teeth, excluding third molars, and presented in a percentage. Diagnosis of periodontal conditions was made following the American Academy of Periodontology (AAP) and European Federation of Periodontology (EFP) Workshop Classification 2017 (25). Additional data were gathered regarding participants' smoking status, presence of comorbidities such as diabetes mellitus (DM) and hypertension, oral hygiene practices, and regularity of dental visits. These additional data were obtained through patient self-reports via medical records from both hospitals, with each variable marked by an identifiable personal code. However, information on other potentially relevant variables—such as educational level, socioeconomic status, and the use of adapted oral hygiene devices—was not available in our dataset. To ensure objective findings, these self-reported data were collected prior to the clinical examination. Furthermore, the clinical examiner was blinded to all responses throughout the periodontal assessment.

### 2.3 Manual dexterity measurement

The manual dexterity test (MPUT) was performed as follows: twelve objects were randomly distributed on a wooden table adjacent to a container. Objects included a wing nut, bolt, key, nail, two sizes of Indonesian coins, washer, safety pin, paper clip, large and medium-sized hexagonal nuts, and a small square nut. Participants were situated in front of a wooden table positioned on a table aligned lengthwise. Test objects were all positioned on the table on the same side as the hand being tested, while the container was situated on the opposite side. Participants were required to pick up individual objects as quickly as possible and place them into a container with a 6-inch diameter. A stopwatch was used to record the exact time taken for each attempt to measure performance speed (26, 27) (see Supplementary Figures 1, 2). The examiner was blinded to any prior periodontal data.

### 2.4 Statistical analyses

Data analysis was performed using IBM SPSS Statistics, version 29.0 (IBM Corp., Armonk, NY, USA), with statistical significance set at *p* < 0.05. Data normality was assessed using the Kolmogorov–Smirnov test. Descriptive statistics included means, standard deviations, medians with interquartile range (IQR), and frequencies for categorical variables. Comparisons between groups with normally distributed data (plaque score) were performed using the independent t-test, while non-normally distributed variables (MPUT) were analyzed using the Mann–Whitney U test. Categorical variables were compared using the Chi-Square test, with Yates' continuity correction or Fisher's exact test applied as appropriate. Correlation between plaque score and manual dexterity was examined using Spearman's rho correlation. For multivariable logistic regression analysis, the data were categorized based on the median value. The primary outcome of this study was plaque score, as it directly reflects oral hygiene performance and plaque accumulation, which were hypothesized to be influenced by impaired manual dexterity in patients with RA. Intra-examiner reliability of clinical measurements was evaluated using ICC prior to the primary analysis.

## 3 Results

### 3.1 Characteristics of the study population

A total of 108 patients were screened for eligibility. Of these, 27 were excluded for various reasons, including healthy gingival status, unwillingness to participate, and overlapping autoimmune conditions such as systemic lupus erythematosus (SLE) and psoriatic arthritis. One participant was excluded due to being unable to perform the motoric test. The final patients included in this study were 80 (as shown in Figure 1).

Table 1 summarizes the characteristics and comorbidities of the 80 participants enrolled in the study, comprising 40 RA cases and 40 non-RA controls. When compared to the non-RA group, individuals with RA showed significant differences in demographic and clinical characteristics, including a predominance of females (*p* = 0.002); older age [49.50 [19.75] vs. 39.50 [20.75]], *p* = 0.002); fewer regular dental visits (*p* = 0.04); and higher frequency of comorbidities such as diabetes mellitus (*p* = 0.02) and hypertension (*p* = 0.03). Smoking habits, alcohol consumption, oral hygiene practices, and BMI were comparable between the RA and non-RA groups. The majority of participants were right-handed.

**Table 1:** Characteristics and comorbidities in the RA and non-RA groups.

Variable	RA		non-RA		*p*-value
	n	%	n	%	
Gender					
Female (*n* = 64)	38	95.0	26	65.0	
Male (*n* = 16)	2	5.0	14	35.0	0.002^a^
Age (years) Median [IQR]	49.50[19.75]		39.50[20.75]		0.002^b^
Smoking status					
Yes (current smoker)	1	2.5	4	10.0	
No (non-smoker)	39	97.5	36	90.0	0.359^c^
Alcohol consumption					
Yes (current alcohol drinker)	0	0.0	4	10.0	
No (non-alcohol drinker)	40	100.0	36	90.0	0.116^c^
Regular (annually) dental check-up					
Yes	6	15.0	15	37.5	
No	34	85.0	25	62.5	0.042^a^
Daily tooth brushing frequency					
Once a day	1	2.5	0	0.0	
Twice a day	31	77.5	37	92.5	
Thrice a day	8	20.0	3	7.5	0.149^d^
Dental floss/interdental brush using					
Yes	5	12.5	7	17.5	
No	35	87.5	33	82.5	0.754^a^
Diabetes mellitus					
Yes	6	15.0	0	0.0	
No	34	85.0	40	100.0	0.026^c^
Hypertension					
Yes	11	27.5	3	7.5	0.039^a^
No	29	72.5	37	92.5	
BMI (kg/m^2^) [Mean ± SD]	23.51 ± 4.60		24.75 ± 4.50		0.229^e^
Underweight, <18.5	5	12.5	2	5.0	
Normal, 18.5–24.9	21	52.5	18	45.0	
Overweight, 25–29.9	11	27.5	16	40.0	
Obese, > 30	3	7.5	4	10.0	
Dominant hand					
Right	38	95.0	39	97.5	
Left	2	5.0	1	2.5	

a Yate's Correction.

b Mann Whitney.

c Fisher's-Exact Test.

d Chi-square.

e Independent T-test.

Characteristics, disease activity, and treatment of 40 patients with RA who were included in the study are presented in Table 2. The majority were female (95%), with only a small proportion of male participants (5%). Most patients had a disease duration of more than 2 years (87.5%), while a minority had a shorter disease duration (< 2 years, 12.5%). Twenty-five percent of patients have hand deformities. Based on disease activity assessed by DAS-28 ESR (mean ± SD: 3.62 ± 1.24), 25% of patients were in remission, 15% had low disease activity, 50% had moderate disease activity, and 10% had high disease activity. Regarding pharmacological treatment, methotrexate (MTX) monotherapy was the most commonly used disease-modifying anti-rheumatic drug (DMARD) (45%), followed by combination therapies including MTX with sulfasalazine (SSZ) (20%) and MTX with leflunomide (LEF) (7.5%). Other regimens included SSZ (12.5%), hydroxychloroquine (HCQ) (2.5%), SSZ combined with HCQ (10%), and LEF combined with SSZ (2.5%).

**Table 2:** Clinical characteristics, disease activity, and treatment regimens of RA patients.

Variable	*N*	%
Gender		
Female	38	95.0
Male	2	5.0
Duration of disease		
< 2 years	5	12.5
>2 years	35	87.5
Hand deformity		
Yes	10	25.0
No	30	75.0
Level of disease activity DAS28-ESR (Mean ± SD)	(3.62 ± 1.24)	
Remission	10	25.0
Low	6	15.0
Moderate	20	50.0
High	4	10.0
DMARD		
MTX	18	45.0
SSZ	5	12.5
HCQ	1	2.5
MTX + SSZ	8	20.0
MTX + LEF	3	7.5
SSZ + HCQ	4	10.0
LEF + SSZ	1	2.5

### 3.2 Clinical periodontal parameter of periodontitis patients

Periodontal diagnoses, FMBS, and plaque score in RA and non-RA groups are presented in Table 3. In the RA group, 15% of patients were diagnosed with gingivitis, 12.5% with stage 1–2 periodontitis, and 72.5% with stage 3–4 periodontitis. In contrast, in the non-RA group, 55% presented with gingivitis, 2.5% with stage 1–2 periodontitis, and 42.5% with stage 3–4 periodontitis. The difference between groups was statistically significant (*p* = 0.001). The number of stage 3 and 4 periodontitis cases was higher in the RA group compared to the non-RA group (*n* = 29 vs. *n* = 17). Overall, periodontal diagnoses differed significantly (*p* = 0.001) between the two groups. There was no significant difference in FMBS between the two groups; however, plaque score showed a significant difference, with the RA group exhibiting a higher plaque score (*p* = 0.036). Table 4 presented the periodontal status of periodontitis patients in this study. Regarding quantitative periodontal profiles, the non-RA cohort exhibited numerically higher median values for PD, CAL, and deepest CAL compared to the RA cohort; however, these variances did not reach statistical significance (PD: *p* = 0.178, CAL: *p* = 0.201, deepest CAL: *p* = 0.219). Conversely, a markedly greater burden of localized destruction was observed in the non-RA group, characterized by a significantly higher number of sites with PD ≥ 5 mm (*p*  = 0.001) and a substantially greater proportion of mobile teeth (*p* = 0.015). Meanwhile, the proportion of missing teeth remained statistically comparable between the two groups (*p* = 0.218).

**Table 3:** Periodontal diagnoses, FMBS, and plaque scores in the RA and non-RA groups.

Variable	*N*	%	*p*-value
Periodontal diagnoses			
Gingivitis			0.001^a^
RA	6	15.0	
Non-RA	22	55.0	
Stage 1–2 periodontitis			
RA	5	12.5	
Non-RA	1	2.5	
Stage 3–4 periodontitis			
RA	29	72.5	
Non-RA	17	42.5	
FMBS (%) Median [IQR]			
RA	22.86[18.46]		0.055^b^
Non-RA	30.45[28.32]		
Plaque score (full-mouth) (Mean ± SD)			
RA	57.95 ± 31.10		0.036^c^
Non-RA	44.78 ± 23.47		

a Chi-square.

b Mann–Whitney.

c Independent T-test.

**Table 4:** Clinical periodontal parameters in the RA and non-RA groups with periodontitis.

Periodontal parameters	RA (*n* = 34)	non-RA (*n* = 18)	*p*-value
	Median [IQR]	Median [IQR]	
PD (mm)	2.24[0.91]	2.55[1.22]	0.178^a^
CAL (mm)	2.84[1.38]	3.47[2.25]	0.201^a^
Deepest CAL (mm)	6.50[3.00]	8.00[3.75]	0.219^a^
NPD ≥ 5 mm	2.00[8.25]	8.50[19.25]	0.001^a^
Presence of mobile teeth, *n* (%)	5.9	33.3	0.015^b^
Presence of missing teeth, *n* (%)	91.2	77.8	0.218^b^

a Mann–Whitney.

b Chi-square.

### 3.3 Manual dexterity

RA patients demonstrated significantly poorer manual dexterity than controls across all MPUT conditions. The median value time (in seconds) were consistently longer in the RA group for both the dominant and non-dominant hands under eyes-open conditions (DHEO: 15.81 vs. 11.83; NdHEO: 17.28 vs. 13.27; both *p* < 0.001). This difference persisted under eyes-closed conditions, with RA patients showing markedly prolonged times for the dominant hand (DHEC: 24.91 vs. 18.50; *p* < 0.001) and non-dominant hand (NdHEC: 25.97 vs. 22.93; *p* = 0.005) (Table 5).

**Table 5:** MPUT performance in the RA and non-RA groups.

MPUT subtests (s)	RA (*n* = 40)	non-RA (*n* = 40)	*p*-value
	Median [IQR]	Median [IQR]	
MPUT (s)			
DHEO	15.81[6.64]	11.83[4.10]	<0.001
NdHEO	17.28[6.06]	13.27[4.12]	<0.001
DHEC	24.91[11.76]	18.50[7.36]	<0.001
NdHEC	25.97[9.72]	22.93[8.52]	0.005

Mann–Whitney.

### 3.4 Correlation between manual dexterity and plaque

Table 6 summarizes the correlations between MPUT subtests, and plaque score. Within the RA group, analyses identified significant associations between non-dominant hand eyes open (NdHEO) [r = 0.430, 95% CI (0.14–0.67), *p* = 0.006] and non-dominant hand eyes closed (NdHEC) [r = 0.435, 95% CI (0.14–0.67), *p* = 0.005] with plaque scores. In contrast, the non-RA group showed no significant associations between any MPUT subtests and plaque.

**Table 6:** Correlation between MPUT subtests and plaque scores in the RA and non-RA groups.

MPUT subtests (s)	RA (*n* = 40)			non-RA (*n* = 40)		
	r	*p*-value	95% CI	r	*p*-value	95% CI
DHEO	0.157	0.334	[−0.17–0.46]	0.211	0.191	[−0.17–0.46]
NdHEO	0.430	0.006^a^	[0.14–0.67]	0.061	0.711	[−0.24–0.37]
DHEC	0.191	0.237	[−0.14–0.50]	0.173	0.285	[−0.13–0.45]
NdHEC	0.435	0.005^a^	[0.14–0.67]	0.210	0.193	[−0.10–0.49]

Spearman's rho correlation.

a Significant at the level < 0.05.

We performed a multivariable logistic regression analysis to test whether manual dexterity remains independently associated with plaque after controlling for age, DM, hypertension, regular dental check-ups (Table 7). The result showed that NdHEC was significantly associated with the plaque (*p* = 0.036), with individuals in the better NdHEC (< 24 s) had a significantly lower likelihood of having poor plaque [OR = 0.033; 95% CI (0.001–0.797)].

**Table 7:** Multivariable logistic regression analysis of factors associated with plaque accumulation after adjustment for Age, comorbidities, and MPUT in the RA group.

Variable	B	SE	*p*-value	OR (95% CI)
Age (ref. to < 49.5)	−0.878	0.877	0.317	0.42 (0.07–2.32)
Diabetes mellitus (ref. to no)	−1.287	1.269	0.310	0.28 (0.02–3.32)
Hypertension (ref. to no)	−0.788	1.269	0.420	0.46 (0.07–3.09)
MPUT				
NdHEC (ref. to < 26 s)	−3.416	1.627	0.036^a^	0.03 (0.001–0.80)
DHEO (ref. to < 16 s)	2.175	1.182	0.066	8.80 (0.87–89.24)
NdHEO (ref. to < 22 s)	0.480	1.355	0.723	1.62 (0.11–22.98)

a Significant at the level < 0.05.

## 4 Discussion

This study was conducted to investigate the relationship between manual dexterity and plaque accumulation of rheumatoid arthritis patients in an Indonesian cohort. Ninety-five percent of RA participants were female, which is consistent with previous epidemiological evidence. Numerous studies have reported that females are more likely to develop RA than males (1, 28–30). This gender distribution observed in our sample therefore reflects the established demographic pattern of RA prevalence. Participants with RA in this study were, on average, older than those without RA. This result aligns with earlier studies indicating that RA most commonly develops between the ages of 40 and 60 (1, 31). According to Indonesia's 2018 National Basic Health Research (RISKESDAS 2018) (32), the highest prevalence of RA and other joint diseases was observed among individuals over the age of 75, suggesting that while RA typically manifests in middle age, its burden may increase substantially in later life. Nevertheless, the specific age distribution within our study cohort might possess limited generalizability to the wider population. Beyond demographic considerations, lifestyle factors such as smoking and alcohol consumption are well-established shared risk factors for both RA and periodontal diseases (10). In the present study, no significant differences in smoking habits and alcohol consumption were observed between RA and non-RA participants. This finding in smoking habits and alcohol use between RA and non-RA participants may be explained by cultural factors.

Eighty percent of participants from the RA and non-RA groups in this study were female, and in Indonesia, female smoking and drinking alcohol remain uncommon since they carry a negative image. This finding may reflect cultural or lifestyle factors within the study population. Regular annual dental check-ups were more frequently reported by non-RA participants compared with those in the RA group, consistent with findings from a previous study (24). This may indicate that individuals with RA face barriers to accessing routine dental care, potentially due to physical limitations, disease burden, or reduced prioritization of oral health in the context of chronic illness. In addition, the non-RA participants were recruited from a dental hospital, which suggests that these participants may have had better knowledge and awareness regarding oral health, including the importance of regular dental check-ups.

DM and hypertension were reported frequently in the RA group compared to the non-RA group in this study. This is consistent with a previous report that suggested RA individuals are 23% more likely to develop type 1 and 2 diabetes (33), hypertension, and metabolic syndrome (34, 35). Inflammation plays a key role in the development of diabetes and hypertension in RA. Cytokines have an association with insulin resistance and linking with type 2 DM, as well as arterial stiffness and endothelial dysfunction potentially contributing to the development of hypertension (36–38). This study did not specifically investigate the mechanistic links between these comorbidities and periodontal outcomes; therefore, these relationships remain unexamined in our cohort and warrant further investigation.

**Figure 2:**
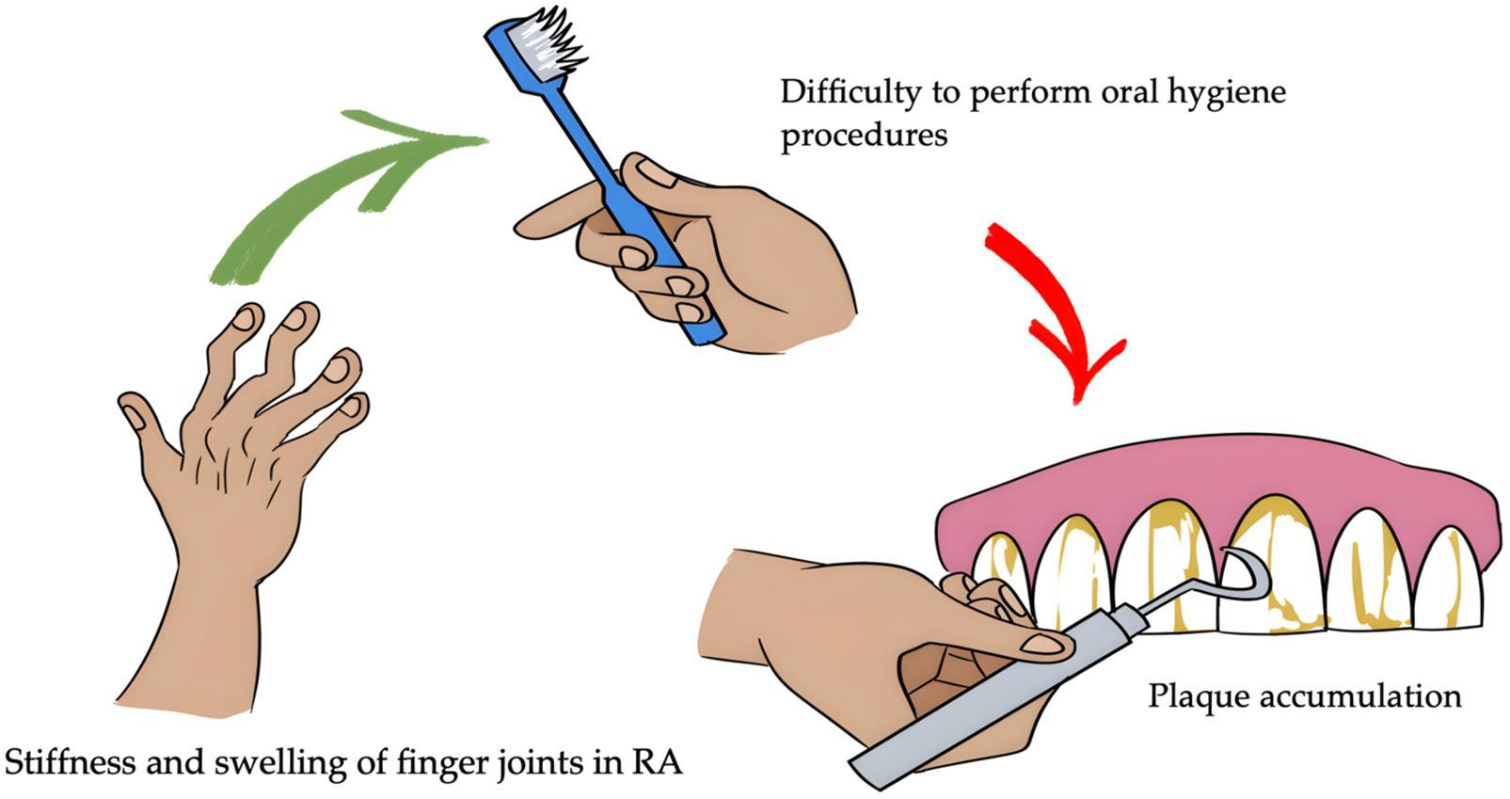
Lower manual dexterity may be associated with plaque accumulation in patients with RA.

This study demonstrated that participants in the RA group more frequently had periodontitis rather than gingivitis when compared to the non-RA group. This finding is consistent with a previous study reporting that RA patients have a greater risk of developing periodontitis compared with the non-RA individuals and a higher number of periodontitis cases (10, 39–41). In contrast, prior studies found that no significant differences of periodontitis case in RA and systemically healthy control groups (42, 43). Variations in these results may reflect differences in study populations, sample sizes, and periodontitis diagnostic criteria. Additionally, stage 3 and 4 periodontitis was most commonly observed among RA subjects in the current cohort. Previous research has similarly reported that moderate to severe periodontitis is highly prevalent in RA patients (28, 41, 44), which aligns with our findings. It is hypothesized that persistent inflammation as well as specific bacteria associated with periodontitis, such as *P. gingivalis*, contribute to the development of RA through PPAD, which is considered to stimulate the production of autoantibodies associated with RA (44, 45). This study also revealed that the RA group exhibited higher plaque scores compared to the non-RA group. Consistent with our research, prior studies have demonstrated similar findings (14, 39, 46). This finding may be associated with the clinical manifestations of RA, such as joint stiffness and swelling (Figure 2), which may limit the manual precision required for effective oral hygiene procedures, and poor oral hygiene has been proposed as a potential link between RA and periodontitis (39, 47). Therefore, the ability of RA patients to perform dental hygiene should be evaluated while examining the pathogenic relationship between RA and periodontitis.

Although several studies have reported more severe periodontal destruction in patients with RA than in controls (12, 48), others have reported contrasting findings. Posada-López et al. found that clinical periodontal parameters, including PD, CAL, and the number of sites with PD > 5 mm, were significantly greater among non-RA patients with periodontitis than among those with RA (49). A similar trend was observed in the present study, with the non-RA group demonstrating higher median PD and CAL values, as well as greater deepest CAL values, although these differences did not reach statistical significance. One possible explanation relates to the different recruitment settings. The non-RA group may not represent a population with optimal oral health, as participants were recruited from a dental hospital, where individuals often seek treatment for advanced and symptomatic periodontal disease. Previous studies have shown that many individuals delay seeking dental care until symptoms become sufficiently severe to warrant professional intervention (50), which may have contributed to the more advanced clinical periodontal manifestations observed in the non-RA group. In addition, prior research has suggested that individual variations in host immune susceptibility, such as interleukin-1 (IL-1) polymorphisms, may independently drive a hyper-inflammatory host response and contribute to aggressive tissue damage regardless of systemic autoimmune status (51). Although genetic susceptibility was not analyzed in the present study, these contextual and biological considerations highlight that periodontal outcomes are influenced by multiple factors. Accordingly, the periodontal findings should be interpreted in light of the study population, recruitment strategy, and the broader literature. Taken together, these findings suggest that factors beyond plaque accumulation may contribute to periodontal status in patients with RA.

In addition to contextual and biological influences, pharmacological factors should also be considered, as medications frequently prescribed to RA patients, such as methotrexate (MTX) and leflunomide (LEF), may have contributed to the observed outcomes. In this study, 45% of the RA patients received MTX monotherapy, while 7.5% were treated with a combination of MTX and LEF. Furthermore, a disease duration of more than 2 years was observed in 87.5% of the patients, reflecting a prolonged exposure to these RA medications which may directly influence periodontal manifestations. Mechanistically, MTX interferes with folic acid metabolism and suppresses the growth of lymphocytes producing TNF-α and IL-6 (52). Previous literature reported that RA patients with periodontitis treated with DMARDs—such as MTX and LEF with or without glucocorticoids—exhibited significant reductions in periodontal parameters, including bleeding on probing (BOP) and PD, alongside decreased salivary levels of IL-6, IL-8, IL-17A, and TNF-α (53). Similarly, Kordtabar et al. found that gingival inflammation, as measured by the gingival index (GI), was higher in RA patients with no history of DMARD consumption, demonstrating the positive impact of these medications in controlling localized inflammation (39). Concurrently, the use of anti-rheumatic medications in our RA cohort may have prevented localized periodontal lesions from progressing into extreme clinical measurements, thereby leveling the numerical outcomes with those of the non-RA group. The potential effects of RA medications on periodontal manifestations were not analytically assessed in this study. As such, any interpretation regarding their role remains speculative and represents a limitation of the present analysis.

FMSs assessed using the MPUT were done under two conditions, eyes open and eyes closed. When administered with eyes open (DHEO and NdHEO), the MPUT can be used to assess adherence to manual precision, whereas the eyes-closed MPUT (DHEC and NdHEC) serves to evaluate the sensory function of the hand control (54). This study revealed statistically significant differences between the two groups, with the RA group demonstrating a longer average task completion time, thereby indicating reduced hand dexterity. When compared with the normative MPUT values reported by Amirjani et al. (27), female participants in the RA group required an average of five seconds longer to complete the task with eyes open and 6–8 s longer with eyes closed.

From a clinical perspective, reduced manual coordination and fine motor control may be associated with a failure to perform interdental cleansing and improper toothbrushing techniques, which could contribute to greater plaque accumulation (55). Aligning with this clinical rationale, our multivariable analysis revealed a significant relationship between NdHEC and plaque after adjusting for age, diabetes mellitus, and hypertension in this study. Although significant correlations were observed in the non-dominant hand, previous studies have reported no consistent differences in functional grip capability between dominant and non-dominant hands in patients with RA, despite greater strength in the dominant hand (56, 57). This suggests that while hand dominance may influence general hand function, fine motor deficits in the non-dominant hand can still be clinically relevant, particularly for tasks requiring precision, such as oral hygiene procedures. Conversely, alternative evidence indicates that the dominant hand can present as up to 20% weaker than the non-dominant hand in certain RA cohorts (58). This divergence emphasized the importance of assessing both hands individually when evaluating manual dexterity and its potential impact on activities of daily living. While previous studies have reported a tendency of association between reduced manual dexterity and age (17, 59), our investigation did not corroborate this finding. This discrepancy may be attributed to differences in age distribution, as earlier studies primarily included older populations (> 60 years) and variations in systemic health conditions.

To our knowledge, this is the first study to investigate the association between manual dexterity and plaque accumulation in RA patients in Indonesia. The observed relationship between these factors may have important clinical implications, as lower FMSs are independently associated with greater plaque accumulation. Nevertheless, this association does not permit firm conclusions regarding the direct impact of manual dexterity on the severity of periodontal disease. From a clinical perspective, RA patients may benefit from tailored oral hygiene strategies, such as the use of electric toothbrushes or ergonomically designed devices to support effective plaque control. These findings also highlight the importance of interdisciplinary collaboration between rheumatologists and periodontists to support comprehensive care addressing both systemic and oral health.

This study has several limitations. First, given that RA is characteristically more common in females, we were unable to match the groups based on sex, resulting in a higher prevalence of female participants. Second, participants were recruited from different clinical settings, which may have introduced selection bias and contributed to baseline differences between the groups. Therefore, the findings may not be directly generalizable to populations with different demographic or healthcare characteristics. Third, although smoking status, oral hygiene habits, dental attendance, and comorbidities were recorded, other potentially relevant variables, including educational level, socioeconomic status, and the use of adapted oral hygiene devices, were not collected and therefore could not be evaluated in the present study. Finally, due to the cross-sectional design, the observed association between manual dexterity and plaque accumulation should not be interpreted as causal.

To overcome these limitations, future longitudinal studies should aim to include a greater number of participants to enhance the generalizability of the findings. Comprehensive investigations are also needed to explore the complex interaction of factors affecting periodontal status, RA disease activity, medication, and manual dexterity. By addressing these aspects, future research can provide a deeper understanding of the mechanisms linking RA and periodontal disease, thereby informing more effective preventive and therapeutic strategies.

## Data Availability

The original contributions presented in the study are included in the article/Supplementary Material, further inquiries can be directed to the corresponding authors.

## References

[1] González-Febles J Sanz M. Periodontitis and rheumatoid arthritis: what have we learned about their connection and their treatment? Periodontol. 2000. (2021) 87(1):181–203. 10.1111/prd.12385 34463976

[2] Ghorbani M Khoshdoozmasouleh N. Distinct oral DNA viral signatures in rheumatoid arthritis: a pilot study. J. Oral Microbiol. (2024) 16(1):2348260. 10.1080/20002297.2024.2348260 38698892 PMC11064737

[3] Almutairi K Nossent J Preen D Keen H Inderjeeth C. The global prevalence of rheumatoid arthritis: a meta-analysis based on a systematic review. Rheumatol. Int. (2021) 41(15):863–77. 10.1007/s00296-020-04731-0 33175207

[4] Deane KD Demoruelle MK Kelmenson LB Kuhn KA Norris JM Holers VM. Genetic and environmental risk factors for rheumatoid arthritis. Best Pract. Res. Clin. Rheumatol. (2017) 31(1):3–18. 10.1016/j.berh.2017.08.003 29221595 PMC5726551

[5] Scher JU Littman DR Abramson SB. Microbiome in inflammatory arthritis and human rheumatic diseases. Arthritis and Rheumatology. (2016) 68(1):35–45. 10.1002/art.39259 26331579 PMC4789258

[6] Astuti L Masulili SLC Gunardi I Sulijaya B Soeroso Y. Periodontal pathogens correlate with rheumatoid arthritis disease parameters: a systematic review based on clinical studies. Dent. J. (Basel). (2025) 13(5):214. 10.3390/dj13050214 40422634 PMC12110451

[7] Kwon TH Lamster IB Levin L. Current concepts in the management of periodontitis. Int. Dent. J. (2021) 71(6):462–76. 10.1111/idj.12630 34839889 PMC9275292

[8] Costalonga M Thumbigere-Math V Herzberg MC. Autoimmunity and periodontitis. J. Periodontal Res. (2026) 0:1–22. 10.1111/jre.70058 41498346

[9] Ding Q Hu W Wang R Yang Q Zhu M Li M. Signaling pathways in rheumatoid arthritis: implications for targeted therapy. Signal Transduct Target Ther. (2023) 8(1):68. 10.1038/s41392-023-01331-9 36797236 PMC9935929

[10] Fuggle NR Smith TO Kaul A Sofat N. Hand to mouth: a systematic review and meta-analysis of the association between rheumatoid arthritis and periodontitis. Front. Immunol. (2016) 2(7):80. 10.3389/fimmu.2016.00080 PMC4774606 26973655

[11] Rovas A Puriene A Punceviciene E Butrimiene I Stuopelyte K Jarmalaite S. Associations of periodontal Status in periodontitis and rheumatoid arthritis patients. J Periodontal Implant Sci. (2021) 51(2):124–34. 10.5051/jpis.2006060303 33913635 PMC8090795

[12] Schmickler J Rupprecht A Patschan S Patschan D Müller GA Haak R. Cross-sectional evaluation of periodontal Status and microbiologic and rheumatoid parameters in a large cohort of patients with rheumatoid arthritis. J. Periodontol. (2017) 88(4):368–79. 10.1902/jop.2016.160355 27858553

[13] Almasi S K Sabbagh M Barzi D Tahooni A Atyabi H B Shabestari S. Relationship between clinical and laboratory findings of rheumatoid arthritis patients with their oral status and disease activity. Caspian J Intern Med. (2021) 12(1):22–8. 10.22088/cjim.12.1.22 33680394 PMC7919168

[14] Pischon N Pischon T Kröger J Gülmez E Kleber B-M Bernimoulin J-P. Association among rheumatoid arthritis, oral hygiene, and periodontitis. J. Periodontol. (2008) 79(6):979–86. 10.1902/jop.2008.070501 18533773

[15] Hashimoto H Hashimoto S Shimazaki Y. Functional impairment and periodontitis in rheumatoid arthritis. Int. Dent. J. (2022) 72(5):641–7. 10.1016/j.identj.2022.01.002 35241287 PMC9485534

[16] Macote-Orosco L Martín-Vacas A Paz-Cortés MM Mourelle Martínez MR De Nova MJ. The relationship between manual dexterity and toothbrushing efficiency in preschool children: a crossover study. Children. (2024) 11(12):1498. 10.3390/children11121498 39767927 PMC11674593

[17] Shin NR Choi JS. Manual dexterity and dental biofilm accumulation in independent older adults without hand disabilities: a cross-sectional study. Photodiagnosis Photodyn. Ther. (2019) 25:74–83. 10.1016/j.pdpdt.2018.11.007 30439530

[18] Lorenz A Tomschi F Schmidt A Stephan H Wiese J Hilberg T. The psychometric properties of the moberg pick-up test (MPUT) to assess fine motor skills in adults with haemophilia. Healthcare (Switzerland). (2026) 14(3):368. 10.3390/healthcare14030368 PMC12897628 41682217

[19] Amirjani N Ashworth NL Olson JL Morhart M Chan KM. Discriminative validity and test-retest reliability of the dellon-modified moberg pick-up test in carpal tunnel syndrome patients. J Peripher Nerv. Syst. (2011) 16(1):51–8. 10.1111/j.1529-8027.2011.00312.x 21504503

[20] Coppers B Heinrich S Tascilar K Phutane U Kleyer A Simon D. Sensor-Assessed grasping time as a biomarker of functional impairment in rheumatoid arthritis. Sci. Rep. (2025) 15(1):6018. 10.1038/s41598-025-90295-7 39972020 PMC11840059

[21] Felder R James K Brown C Lemon S Reveal M. Dexterity testing as a predictor of oral care ability. J. Am. Geriatr. Soc. (1994) 42(10):1081–6. 10.1111/j.1532-5415.1994.tb06213.x 7930333

[22] Oliveira SC Slot DE Celeste RK Abegg C Keijser BJF Van Der Weijden FA. Correlations between two different methods to score bleeding and the relationship with plaque in systemically healthy young adults. J. Clin. Periodontol. (2015) 42(10):908–13. 10.1111/jcpe.12435 26212602

[23] Mühlberg S Jäger J Krohn-Grimberghe B Patschan S Mausberg RF Schmalz G. Oral health-related quality of life depending on oral health in patients with rheumatoid arthritis. Clin. Oral Investig. (2017) 21(9):2661–70. 10.1007/s00784-017-2068-4 28190151

[24] Rodríguez-Lozano B González-Febles J Garnier-Rodríguez JL Dadlani S Bustabad-Reyes S Sanz M. Association between severity of periodontitis and clinical activity in rheumatoid arthritis patients: a case-control study. Arthritis Res. Ther. (2019) 21(1):27. 10.1186/s13075-019-1808-z 30658685 PMC6339403

[25] Tonetti MS Greenwell H Kornman KS. Staging and grading of periodontitis: framework and proposal of a new classification and case definition. J. Clin. Periodontol. (2018) 89(Supp1):S159–72. 10.1111/jcpe.12945 29926952

[26] Armanini KK Weber FM Muraro CF Borges Junior NG Domenech SC Gevaerd MS. Evaluation of manual dexterity in individuals with rheumatoid arthritis. Acta Fisiátrica. (2015) 22(4):166–71. 10.5935/0104-7795.20150032

[27] Amirjani N Ashworth NL Gordon T Edwards DC Chan KM. Normative values and the effects of age, gender, and handedness on the moberg pick-up test. Muscle Nerve. (2007) 35(6):788–92. 10.1002/mus.20750 17326120

[28] Fei C Eriksson K Fei G Delgado Zambrano LF Syed S Lindström C. Comparative analysis of salivary and serum inflammatory mediator profiles in patients with rheumatoid arthritis and periodontitis. Mediators Inflamm. (2025) 20(2025):7739833. 10.1155/mi/7739833 PMC11949604 40151315

[29] Hahn J Malspeis S Choi MY Stevens E Karlson EW Lu B. Association of healthy lifestyle behaviors and the risk of developing rheumatoid arthritis among women. Arthritis Care Res. (Hoboken). (2023) 75(2):272–6. 10.1002/acr.24862 35040282 PMC9289074

[30] Furuya T Inoue E Tanaka E Maeda S Ikari K Taniguchi A. Age and female gender associated with periodontal disease in Japanese patients with rheumatoid arthritis: results from self-reported questionnaires from the IORRA cohort study. Mod. Rheumatol. (2020) 30(3):465–70. 10.1080/14397595.2019.1621461 31116056

[31] Dolcezza S Flores-Fraile J Lobo-Galindo AB Montiel-Company JM Zubizarreta-Macho Á. Relationship between rheumatoid arthritis and periodontal disease—systematic review and meta-analysis. J. Clin. Med. (2025) 14(1):10. 10.3390/jcm14010010 PMC11720692 39797091

[32] Kementerian Kesehatan RI. Laporan Riskesdas Nasional 2018: Penyakit Sendi. Jakarta, Indonesia: Kementerian Kesehatan RI (2018).

[33] Ramamoorthy P Ramamoorthy S Santhosh T Samayan K. A case report of auditory and vestibular findings in a patient with rheumatoid arthritis and diabetes Mellitus. Indian J Otolaryngol Head Neck Surg. (2024) 76(4):3656–60. 10.1007/s12070-024-04668-x 39130318 PMC11306714

[34] Barry S Sheng E Baker JF. Metabolic consequences of rheumatoid arthritis. Arthritis Care Res. (Hoboken). (2025) 77(10):1167–74. 10.1002/acr.25537 40176397 PMC12500507

[35] Samborska-Mazur J Sikorska D Wyganowska-Świątkowska M. The relationship between periodontal Status and rheumatoid arthritis. Systematic Review. Reumatologia. (2020) 58(4):236–42. 10.5114/reum.2020.98436 32921831 PMC7477472

[36] Tian Z McLaughlin J Verma A Chinoy H Heald AH. The relationship between rheumatoid arthritis and diabetes mellitus: a systematic review and meta-analysis. Cardiovasc. Endocrinol. Metab. (2021) 10(2):125–31. 10.1097/XCE.0000000000000244 34124603 PMC8189616

[37] Moller DE. Potential role of TNF-alpha in the pathogenesis of insulin resistance and type 2 diabetes. Trends Endocrinol Metab. (2000) 11(6):212–7. 10.1016/s1043-2760(00)00272-1 10878750

[38] Manavathongchai S Bian A Rho YH Oeser A Solus JF Gebretsadik T. Inflammation and hypertension in rheumatoid arthritis. Journal Rheumatol. (2013) 40(11):1806–11. 10.3899/jrheum.130394 PMC3818311 23996293

[39] Kordtabar S Aghaie M Fakhari E Ali Vakili M Author C. Periodontal condition in patients with rheumatoid arthritis: effect of anti-rheumatic drugs. J Dent Shiraz Univ Med Sci. (2019) 20(3):190–4. 10.30476/DENTJODS.2019.44914 PMC6732181 31579694

[40] Tamášová M Macejová Ž Dorko E Timková S Rimárová K Diabelková J. Oral health and rheumatoid arthritis: a case control study. Cent. Eur. J. Public Health. (2024) 32(Supplement):78–S84. 10.21101/cejph.a7892 39832152

[41] Varshney S Sharma M Kapoor S Siddharth M. Association between rheumatoid arthritis and periodontitis in an adult population-A cross sectional study. J. Clin. Exp. Dent. (2021) 13(10):980–6. 10.4317/jced.57562 PMC8501867 34667492

[42] Rahajoe PS De Smit M Schuurmans G Raveling-Eelsing E Kertia N Vissink A. Increased IgA anti-citrullinated protein antibodies in the periodontal inflammatory exudate of healthy individuals compared to rheumatoid arthritis patients. J. Clin. Periodontol. (2020) 47(5):552–60. 10.1111/jcpe.13277 32141631 PMC7318198

[43] Eriksson K Nise L Kats A Luttropp E Catrina AI Askling J. Prevalence of periodontitis in patients with established rheumatoid arthritis: a Swedish population based case-control study. PLoS One. (2016) 11(5):e0155956. 10.1371/journal.pone.0155956 27203435 PMC4874595

[44] Kurowska W Kuca-Warnawin EH Radzikowska A Maslinski W. The role of anti-citrullinated protein antibodies (ACPA) in the pathogenesis of rheumatoid arthritis. Cent Eur J Immunol. (2017) 42(4):390–8. 10.5114/ceji.2017.72807 29472818 PMC5820977

[45] De Smit MJ Rahajoe PS Raveling-Eelsing E Lisotto P Harmsen HJM Kertia N. Influence of oral Microbiota on the presence of IgA anti-citrullinated protein antibodies in gingival crevicular fluid. Front Oral Health. (2022) 16(3):904711. 10.3389/froh.2022.904711 eCollection 2022 PMC9243218 35784663

[46] Choi IA Kim J-H Kim YM Lee JY Kim KH Lee EY. Periodontitis is associated with rheumatoid arthritis: a study with longstanding rheumatoid arthritis patients in Korea. Korean J Intern Med. (2016) 31(5):977–86. 10.3904/kjim.2015.202 27017391 PMC5016284

[47] Lopez-Oliva I De Pablo P Dietrich T Chapple I. Gums and joints: is there a connection? Part two: the biological link. Br. Dent. J. (2019) 227(7):611–7. 10.1038/s41415-019-0723-7 31605072

[48] Loutan L Alpizar-Rodriguez D Courvoisier DS Finckh A Mombelli A Giannopoulou C. Periodontal Status correlates with anti-citrullinated protein antibodies in first-degree relatives of individuals with rheumatoid arthritis. J. Clin. Periodontol. (2019) 46(7):690–8. 10.1111/jcpe.13117 31025368

[49] Posada-López A Duque JD Pineda-Tamayo RA Bedoya-Giraldo E Botero JE. Lack of association between periodontitis and rheumatoid arthritis. Reumatol. Clin. (2023) 19(3):123–9. 10.1016/j.reuma.2022.03.008 36906387

[50] Rambabu T Koneru S. Reasons for use and nonuse of dental services among people visiting a dental hospital in urban India: a descriptive study. J Educ Health Promot. (2018) 7(1):99. 10.4103/jehp.jehp_193_17 30159345 PMC6088817

[51] Stoupi M Telopoulou M Zisis V Shinas N Emmanouli-Nikoloussi EN. Rheumatoid arthritis and periodontitis: shared pathogenic mechanisms and clinical implications: a narrative review. Oral. (2026) 6(2):40. 10.3390/oral6020040

[52] Seki K Kobayashi A Takahashi N Kamimoto A. Medication-Related osteonecrosis of the jaw in a patient with rheumatoid arthritis with suspected involvement of methotrexate and tocilizumab. J. Dent. Sci. (2024) 19(4):2428–9. 10.1016/j.jds.2024.07.024 39347083 PMC11437342

[53] Kaczyński T Wroński J Głuszko P Kryczka T Miskiewicz A Górski B. Salivary interleukin 6, interleukin 8, interleukin 17A, and tumour necrosis factor *Α*levels in patients with periodontitis and rheumatoid arthritis. Centr Eur J Immunol. (2019) 44(3):269–76. 10.5114/ceji.2019.89601 PMC6953371 31933536

[54] Silva PG Jones A Fernandes ADRC Natour J. Moberg picking-up test in patients with hand osteoarthritis. J Hand Ther. (2017) 30(4):522–8. 10.1016/j.jht.2016.10.005 27863736

[55] Lertpimonchai A Rattanasiri S A-O Vallibhakara S Attia J Thakkinstian A. The association between oral hygiene and periodontitis: a systematic review and meta-analysis. Int. Dent. J. (2017) 67(6):332–43. 10.1111/idj.12317 28646499 PMC5724709

[56] Umay E Çevikol A Çakci A. An evaluation of hand dominancy and its relationship with hand involvement and hand dexterity in patients with rheumatoid arthritis. Archive of Rheumatology. (2013) 28(3):173–80. 10.5606/tjr.2013.2804

[57] Elsaman AM Sayed A Radwan AR. Hand dominance in early and established rheumatoid arthritis: evaluation by dynamometer, ritchie index and musculoskeletal ultrasound: a cross-sectional study. Reumatismo. (2020) 72(3):131–44. 10.4081/reumatismo.2020.1273 33213126

[58] Sferra da Silva G De Almeida Lourenço M De Assis MR. Hand strength in patients with RA correlates strongly with function but not with activity of disease. Adv. Rheumatol. (2018) 58(1):20. 10.1186/s42358-018-0020-1 30657069

[59] Hashimoto H Hashimoto S Muto A Dewake N Shimazaki Y. Influence of plaque control on the relationship between rheumatoid arthritis and periodontal health Status among Japanese rheumatoid arthritis patients. J. Periodontol. (2018) 89(9):1033–42. 10.1002/JPER.17-0575 29763516

